# Redefining the Collagen Composition of Human Fasciae: Emerging Collagen Types and Structural Heterogeneity

**DOI:** 10.3390/ijms27021021

**Published:** 2026-01-20

**Authors:** Caterina Fede, Claudia Clair, Lucia Petrelli, Yunfeng Sun, Carlo Biz, Andrea Angelini, Pietro Ruggieri, Carla Stecco

**Affiliations:** 1 Department of Neuroscience, Institute of Human Anatomy, University of Padova, 35121 Padova, Italy; claudia.clair@unipd.it (C.C.); lucia.petrelli@unipd.it (L.P.); yunfeng.sun@studenti.unipd.it (Y.S.); carla.stecco@unipd.it (C.S.); 2 Padova Neuroscience Center, University of Padova, 35129 Padova, Italy; 3 Department of Orthopedics and Orthopedic Oncology, University of Padova, 35128 Padova, Italy; carlo.biz@unipd.it (C.B.); andrea.angelini@unipd.it (A.A.); pietro.ruggieri@unipd.it (P.R.)

**Keywords:** fascia, collagen, collagen type VI, collagen type XII, extracellular matrix, superficial fascia, deep fascia

## Abstract

Fascia has traditionally been described as a passive connective tissue mainly composed of collagen types I and III. Recent research, however, has revealed its structural and functional complexity, suggesting the possible presence of additional collagen types. This study aimed to quantify the presence and distribution of collagen types I, III, VI, and XII in human superficial and deep fasciae to improve understanding of fascial extracellular matrix composition. Superficial and deep fascia samples were collected from 19 adult patients (ages 20–83 years; thigh and lumbar area). Histology, Azan Mallory staining, hydroxyproline quantification, Western blotting, and immunohistochemistry were performed. The results indicated that deep fascia contained significantly more total collagen than superficial fascia (0.55 ± 0.17 µg/mg vs. 0.36 ± 0.14 µg/mg, *p* < 0.01). Collagen type VI was the most abundant and widely distributed subtype in both superficial and deep fasciae (mean ratio equal to 0.24 ± 0.13 and 0.27 ± 0.10, respectively), nearly double that of collagen types I (0.12 ± 0.07 and 0.11 ± 0.08), III (0.13 ± 0.09 and 0.17 ± 0.11), and XII (0.13 ± 0.11 and 0.13 ± 0.04). Moreover, statistically significant anatomical differences were observed, despite considerable interindividual variability. Fasciae from the thigh showed higher levels of collagen types I and III (mean ratio of 0.17 and 0.27, respectively, in deep fascia; 0.14 for both types in superficial fascia), whereas fasciae of the lumbar region exhibited greater levels of collagen types VI and XII (ratio equal to 0.33 and 0.15, respectively, in deep fascia; 0.36 and 0.20 in superficial fascia). Overall, these findings highlighted the structural complexity and regional specialization of human fasciae, with potential functional implications for mechanotransduction and tissue adaptation.

## 1. Introduction

Traditionally, fascia was regarded as a passive connective tissue layer surrounding muscles and organs, primarily composed of collagen fibers and fibroblasts. In recent years, however, research has demonstrated that fascia is a more complex and functionally active system. Multiple types of fasciae—superficial, deep, visceral, and neural—have been identified, each sharing structural and functional characteristics, while exhibiting distinct cellular organizations, extracellular matrix composition, and associated anatomical structures [1]. These variations reflect the specialized physiological roles performed by each fascial subtype.

The superficial fascia (SF) is characterized by an extracellular matrix (ECM) composed of collagen and elastic fibers in a 1:1 ratio, forming a three-dimensional network in which fibroblasts and immune cells, like mast cells, are embedded [2]. Functionally, it provides mechanical support and protection, while facilitating the proper functioning of vascular and neural structures. Owing to these properties, the SF plays a crucial role in lymphatic drainage, dermatomeric perception cutaneous repair, and the maintenance of a smooth gliding interface between the skin and the underlying muscles [1,2,3].

The deep fascia (DF), instead, consists of two to three distinct layers of dense connective tissue separated by thin layers of loose connective tissue rich in water and glycosaminoglycans (GAGs) [4]. Each dense layer consists of parallel bundles of collagen fibers whose orientations differ between layers by approximately 78° [5], a configuration that contributes to the fascia’s mechanical adaptability. Collagen fibers are synthesized by fibroblasts, and, alongside these cells, specialized cells termed fasciacytes [6] are described, which are responsible for producing hyaluronan. Additionally, elastic fibers account for around 1% of the tissue’s composition, allowing the fascia to withstand stretching and facilitating the efficient distribution of mechanical stresses [2].

Traditionally, studies have focused on the composition of collagen types I and III within the superficial and deep fasciae, as these collagens constitute the principal structural components of human connective tissues [4,7]. Additionally, research has investigated how these collagen types can be altered by various stimuli, including hormonal [8], mechanical [9], and age-related factors [10]. For instance, it has been demonstrated that the total collagen content in the intramuscular connective tissue of elderly men (79.0 ± 12.4 years) is approximately twice that observed in young individuals. Moreover, with advancing age, a significant increase in type I collagen has been reported, reducing fascial adaptability and impairing muscle–fascia interactions [10].

Furthermore, the presence of estrogen receptors in fascial fibroblasts influences the synthesis of type I and type III collagen: in the deep fascia, during the postmenopausal period, fibroblasts exhibit a decreased production of type III collagen accompanied by a simultaneous and significant increase in type I collagen synthesis [8], contributing to fascial rigidity.

Even pathologies, such as obesity [11] and Dupuytren’s disease [12], cause modifications at the fascia level, with significant alterations in total collagen and in type I and III collagens, with a consequent variation in the physiological behavior of the fasciae: the increase in collagen type I has been correlated to a non-physiological fibrosis and stiffness of the tissue, and a parallel reduced capability to adapt to different stimuli [10,12,13].

Nonetheless, the documented heterogeneity of collagen types across different connective tissues has prompted the hypothesis that fasciae may contain additional collagen types beyond type I and III. Indeed, it has been reported that collagen type VI is an important component of the ECM across all human connective tissues [14], where it performs multiple roles, like contributing to the mechanical properties typical of ECM collagens or exerting more specialized cytoprotective functions [15]. It can also bridge the resident cells to the surrounding connective tissue to organize the three-dimensional tissue architecture [15]. The role of collagen type VI in the tendon was confirmed by Cenni et al., who highlighted that this type of collagen is also a critical component for the correct mechanical response of tendon fibroblasts [16].

Collagen type XII instead is associated with type I collagen and is therefore present in the same tissues. Its function modulates tissue flexibility, enhances resistance to stretching, and supports dynamic adaptation to mechanical forces [15,17].

Moreover, both collagens type VI and XII are involved in skin wound repair processes, showing an increase in their expression seven days after injury, with a subsequent progressive reduction [18,19].

In conclusion, given the presence of these types of collagens in connective tissues, in this study, we propose to verify and consequently quantify their presence also in the fasciae (superficial and deep) to better understand the matrix composition of these structures.

## 2. Results

**Figure 1:**
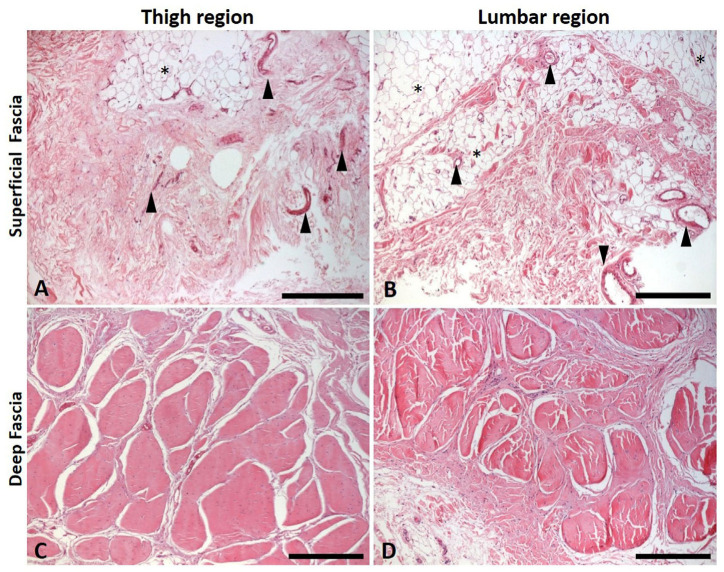
Hematoxylin–eosin staining of a section of superficial (**A**,**B**) and deep fascia (**C**,**D**) of the thigh (**A**,**C**) and of the lumbar region (**B**,**D**). Arrowheads indicate blood vessels; asterisks indicate adipocytes. Scale bars: (**A**–**D**) = 400 µm.

The hematoxylin and eosin stain showed how the superficial (Figure 1A,B) and deep fascia (Figure 1C,D) exhibit distinct organizations of the cellular structure and the extracellular matrix, in the thigh and lumbar regions. The deep fascia is composed of densely packed collagen fibers (Figure 1C,D), whereas the superficial fascia contains loosely arranged collagen and numerous blood vessels and adipocyte-rich areas (Figure 1A,B).

**Figure 2:**
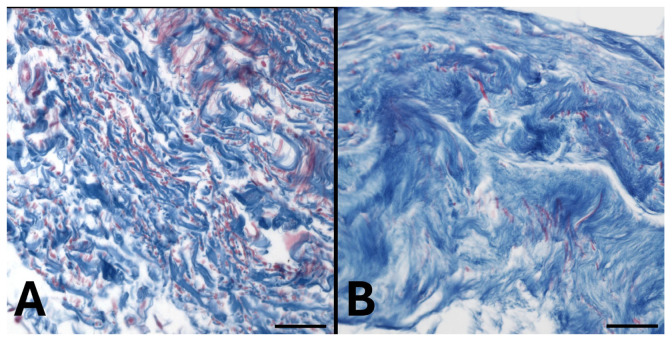
Azan Mallory staining of superficial (**A**) and deep fasciae (**B**) of the hip region, highlighting the presence of collagen fibers (blue). Nuclei of the cells are stained in red. Scale bars = 100 µm.

The distribution of collagen fibers was confirmed by Azan Mallory staining (Figure 2): abundant collagen fibers, stained in blue, were highlighted both in the superficial (Figure 2A) and in the deep (Figure 2B) fasciae.

**Figure 3:**
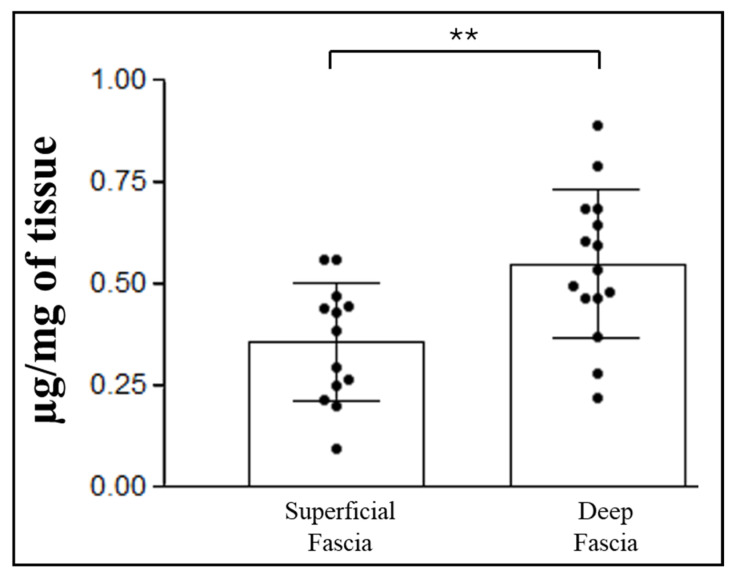
Quantification of hydroxyproline content (µg/mg of tissue) in superficial and deep fasciae. Statistical difference between the two groups were tested by Mann–Whitney test: ** *p* < 0.01.

The total collagen content analysis revealed a significantly (*p* = 0.006) higher amount of collagen in the deep fascia compared to the superficial fascia (Figure 3). Specifically, the superficial fascia contained a mean of 0.36 ± 0.14 µg of hydroxyproline per mg of tissue, whereas the deep fascia showed a mean of 0.55 ± 0.17 µg/mg.

**Figure 4:**
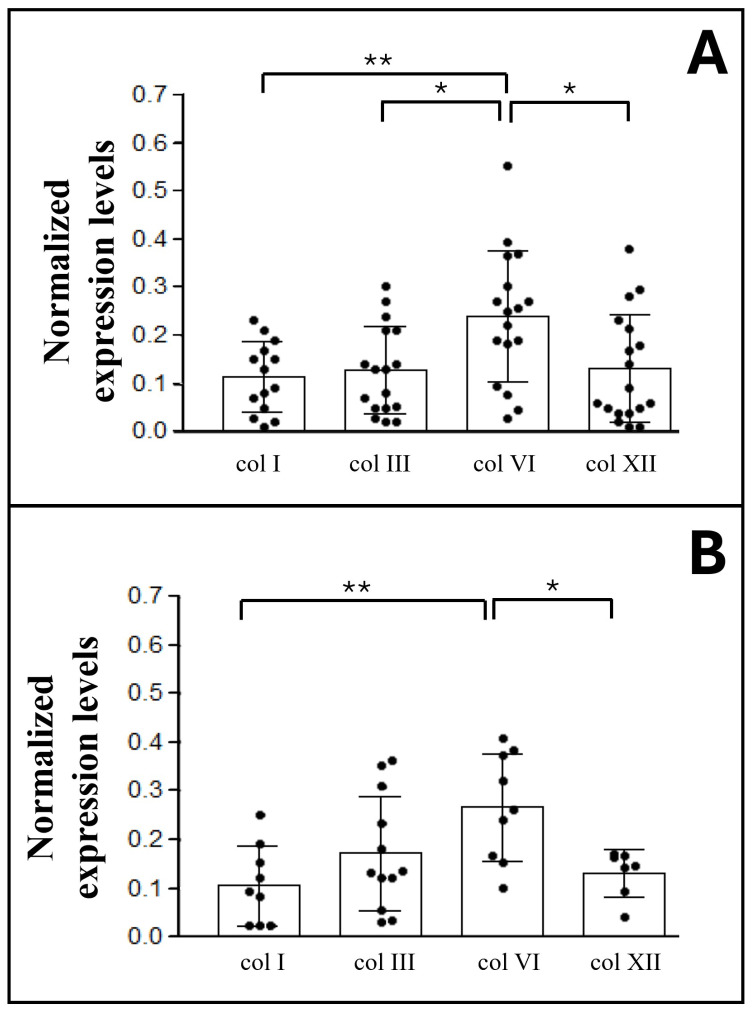
Western blot analysis of collagen subtypes in superficial (**A**) and deep fasciae (**B**). Chemiluminescent signal of col I, col III, col VI, and col XII, normalized with total protein intensities determined by Ponceau-S staining. Statistical differences were tested by Tukey’s test: * *p* < 0.05, ** *p* < 0.01.

Subsequent immunoblot analyses assessed the amount of collagen types I, III, VI, and XII in both superficial (Figure 4A) and deep (Figure 4B) fasciae.

The mean ratio values for collagen types I, III, and XII in the superficial fascia were similar, respectively, 0.12 ± 0.07 (col I), 0.13 ± 0.09 (col III), and 0.13 ± 0.11 (col XII) (Figure 4A). In contrast, collagen type VI was present at approximately twice the amount of the other types, with a mean value of 0.24 ± 0.13, which is statistically significantly different from the other collagen types (I, III, and XII).

In the deep fascia, collagen type VI showed a mean value of 0.27 ± 0.10, (Figure 4B), which is significantly higher than the mean quantity of collagen type I (0.11 ± 0.08, *p* = 0.004) and XII (0.13 ± 0.04).

Collagen type III (mean value = 0.17 ± 0.11) did not show significant differences with other types of collagens present in the deep fascia.

**Figure 5:**
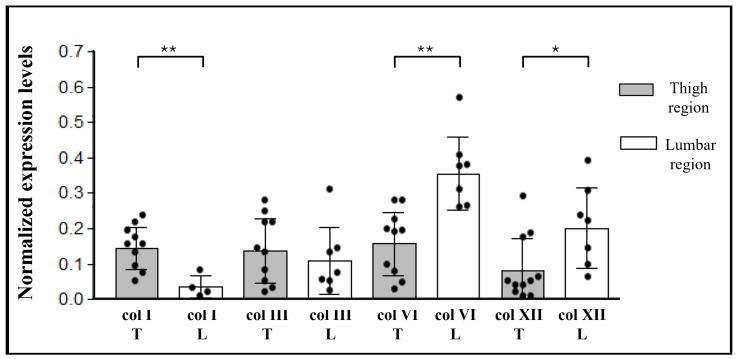
Western blot analysis of collagen subtypes in superficial fascia. Chemiluminescent signal of col I, col III, col VI, and col XII, normalized with total protein intensities determined by Ponceau-S staining, and divided based on the sample origin area. Statistical difference were tested by Mann–Whitney test: * *p* < 0.05, ** *p* < 0.01. T: thigh; L: lumbar region.

**Figure 6:**
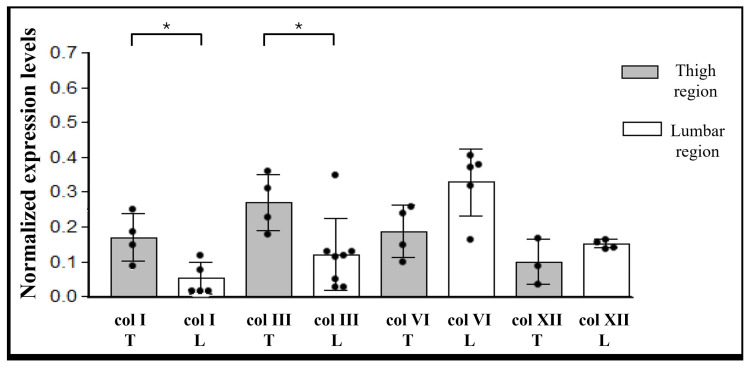
Western blot analysis of collagen subtypes in deep fascia. Chemiluminescent signal of col I, col III, col VI, and col XII, normalized with total protein intensities determined by Ponceau-S staining, and divided based on the sample origin area. Statistical differences were tested by Mann–Whitney test: * *p* < 0.05. T: thigh; L: lumbar region.

Furthermore, samples from different anatomical regions were stratified by anatomical sites to evaluate potential differences (Figure 5 and Figure 6).

Figure 5 shows statistically significant zone-dependent differences for collagens type I, VI, and XII of the superficial fascia. Indeed, collagen type I content was higher in the SF of the thigh (0.14 ± 0.06) compared with the SF of the lumbar region (0.03 ± 0.02). In contrast, both type VI and XII collagens were present in greater amounts in the SF of the back than in the thigh (0.36 ± 0.09 vs. 0.16 ± 0.08 for collagen type VI, and 0.20 ± 0.11 vs. 0.08 ± 0.08 for collagen type XII). Conversely, collagen type III showed similar levels in both regions, with mean values of 0.14 ± 0.08 in the thigh and 0.11 ± 0.08 in the lumbar region.

In the deep fascia, collagen types I and III presented significantly different content between body areas (both *p* = 0.03) (Figure 6): both types were higher in the thigh, with values equal to 0.17 ± 0.06 for the col type I, and 0.27 ± 0.07 for the col type III. Respectively, in the lumbar region the ratios were 0.05 ± 0.04 and 0.12 ± 0.09. Anatomic site-dependent differences were observed also for collagen type VI and type XII, with greater values in the lumbar region with respect to the hip, although not statistically significant: mean values are equal to 0.19 ± 0.06 in the hip region and 0.33 ± 0.08 in the lumbar region for collagen type VI, and 0.10 ± 0.05 and 0.15 ± 0.01, respectively, in the thigh and in the back for collagen type XII.

In general, all collagen subtypes exhibited substantial inter-individual variability, yet the group means differed significantly. This variability persisted when the data were stratified by sex or age, with no significant differences observed between these subgroups (data not shown).

**Figure 7:**
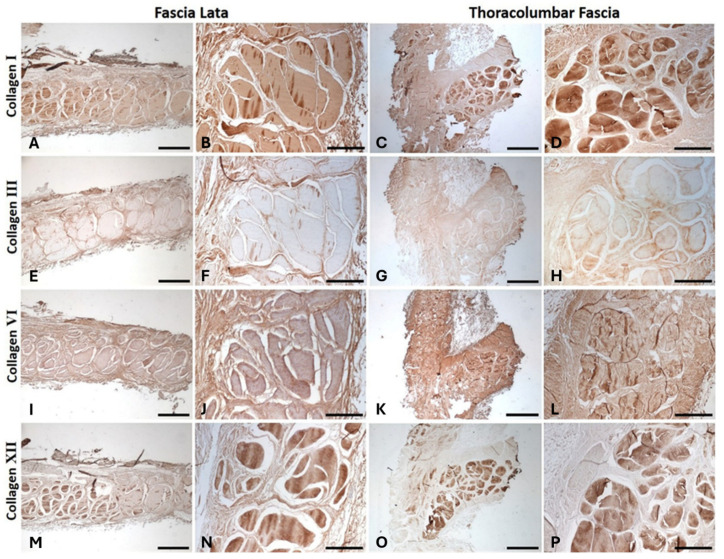
Anti-collagen type I (**A**–**D**), anti-collagen type III (**E**–**H**), anti-collagen type VI (**I**–**L**), and anti-collagen XII (**M**–**P**) staining in the deep fascia of the thigh (fascia lata) (**A**,**B**,**E**,**F**,**I**,**J**,**M**,**N**) and of the back (thoracolumbar fascia) (**C**,**D**,**G**,**H**,**K**,**L**,**O**,**P**). Scale bars: (**A**,**C**,**E**,**G**,**I**,**K**,**M**,**O**) = 1000 µm; (**B**,**D**,**F**,**H**,**J**,**L**,**N**,**P**) = 400 µm.

The presence and distribution of the different types of collagens were confirmed by immunohistochemistry (Figure 7).

The immunostaining confirmed the presence of collagen type I (Figure 7A–D) and collagen type III (Figure 7E–H) fibers in both the fascia lata of the thigh and the thoracolumbar fascia. Type III collagen was predominantly localized toward the outer regions, whereas collagen type I was mainly concentrated toward the central area, corresponding to the more compact zone of the fascia. A strong presence of collagen type VI was also observed (Figure 7I–L), showing a homogeneous distribution throughout the entire fascial tissue. Furthermore, staining revealed the distribution of collagen type XII, predominantly within the dense regions of the deep fascia, closely associated with areas enriched in collagen type I (Figure 7M–P).

## 3. Discussion

Until now, debate about fascia and its collagen composition has generally assumed that these tissues contain mainly collagen types I and III [6]. This study provides, for the first time, evidence of additional collagen types within the fascia, offering a better understanding of the structural and functional complexity of these tissues.

In this study, we demonstrated that the deep fascia contains a significantly greater amount of total collagen compared with the superficial fascia (0.55 ± 0.17 µg/mg and 0.36 ± 0.14 µg/mg, respectively; Figure 3). Moreover, the collagen fibers of the deep fascia are organized into rigid bundles, whereas those of the superficial fascia form a network, confirming that the deep fascia plays a significant role in force transmission, acting as a broad tendon [4], while the superficial fascia likely serves mainly a supportive role for superficial vessels, nerves, and lymphatics [2].

Moreover, the immunoblotting analyses revealed that the two main collagen types typically found in human connective tissue, types I and III, are not the predominant types within fasciae, as previously assumed. Instead, collagen type VI emerged as the most abundant in both superficial and deep fasciae. It is present at approximately twice the level of the other three collagens in both superficial and deep fasciae (Figure 4).

It is already known that collagen type VI works as microfibrillar collagen [16] to bind together various tissue components, such as collagen fibers, nerves, blood vessels, cells, and ground substance, integrating them into a functional and cohesive, yet flexible, connective tissue framework, and helping cells to anchor to the surrounding matrix [14]. Our results of immunostaining demonstrated for the first time the diffuse and uniform distribution of collagen type VI throughout the fascial tissue (Figure 7), suggesting that it could contribute to the mechanical properties of the fasciae, as observed also in the skin [20,21]. Collagen type VI can also stimulate fibroblasts by regulating their migration and proliferation, and it interacts with several membrane receptors involved in intracellular signaling pathways [20,21,22]. This role has already been evidenced in cancer studies, where numerous investigations have shown that collagen type VI contributes to tumor progression and the infiltration into tissues: Gao et al. demonstrated that COL6A1 acts as a master regulator of the basal tumor niche of glioblastoma, where it promotes malignancy and immunosuppression [23]. In bladder cancer, COL6A3 activates the transforming growth factor β (TGF-β) pathway, promoting tumor proliferation and migration and conferring resistance to apoptosis [24].

Furthermore, studies using mouse models of collagen VI-related muscular dystrophies, such as Ullrich congenital muscular dystrophy and Bethlem myopathy, have shown that collagen VI deficiency disrupts the extracellular matrix organization, leading to a compensatory increase in fibrillar collagens, and consequent fibrosis [25,26,27]. Such maladaptive remodeling results in a stiffer and less compliant fascial and muscular connective tissue. In parallel, collagen type VI plays a critical role in skeletal muscle maintenance and regeneration by supporting the satellite cell niche, regulating mechanotransduction, and preserving mitochondrial and autophagic homeostasis [28]. Its absence increases muscle fiber vulnerability to mechanical stress, impairs regenerative capacity, and promotes dysfunctional fibrillogenesis and progressive muscle degeneration [28,29,30]. Considering the substantial inter-individual variability observed in our studies in the amount of the different collagen types, it is plausible that subjects with lower levels of collagen type VI exhibit increased ECM stiffness. This deficiency may compromise normal force transmission and tissue adaptability, thereby predisposing individuals to contractures, reduced range of motion, and early fatigability.

Secondly, we observed collagen type XII (Figure 4 and Figure 7, ratio value = 0.13) in quantities comparable to collagen type I in both superficial and deep fasciae: these results are consistent with the functional role of collagen type XII in regulating collagen type I fibrillogenesis and in modulating tissue flexibility, resistance to stretching, and dynamic adaptation to mechanical forces [17].

In the context of myopathic syndromes, pathogenic variants in the *Col12a1* gene give rise to a recognized subtype known as myopathic Ehlers–Danlos syndrome (EDS), which is characterized by joint hypermobility accompanied by muscle weakness, hypotonia, and impaired motor development [31]. At the tissue level, deficiency or dysfunction of collagen type XII disrupts the orderly assembly of collagen I fibrils, resulting in a disorganized ECM with reduced mechanical efficiency. The identification of collagen type XII in both superficial and deep fasciae in this work also suggests that the fasciae may be altered in EDS. Such alterations could impair force transmission and increase susceptibility to microinstability. A recent work by Wang et al. demonstrated reduced fascial gliding in patients with EDS compared with the control group, indicating that in EDS, fasciae exhibit mechanical incoherence [32]. Specifically, regions of excessive compliance coexist with areas of compensatory stiffness driven by maladaptive remodeling and increased deposition of fibrillar collagens. Clinically, this dual mechanical behavior manifests as the characteristic paradox observed in collagen XII-related EDS, in which joint hypermobility coexists with localized rigidity, myofascial pain, and reduced functional adaptability. The inability of the ECM to appropriately distribute mechanical loads promotes repetitive microtrauma, thereby triggering secondary fibrotic responses that further compromise tissue compliance.

Moreover, in this work, we demonstrated an area-dependent variability in collagen distribution, a feature already highlighted in several previous studies [2,4,14,17]. However, for the first time, we showed that the fasciae of the thigh and lumbar regions exhibit opposite trends in collagen-type content: collagen types I and III are more abundant in the thigh; in contrast, collagen types VI and XII show greater quantities in the lumbar region (Figure 5 and Figure 6). Although superficial fascia has a lower content of total collagen in respect to deep fascia, they show the same trend, influenced by the anatomical location of collagen types I, III, VI, and XII. These findings suggest the high specificity of the fasciae, and that it is not possible to generalize the results of one fascia for all the fasciae of the body. Surely, fasciae form all together a three-dimensional network, but inside it, each fascia of each region has specific features.

The higher abundance of collagen types VI and XII in the thoracolumbar fascia (TLF) provides a plausible molecular basis for its distinct supramolecular organization compared with the fascia lata. Collagen type XII regulates fibrillogenesis and fibril alignment, while collagen type VI stabilizes the inter-fibrillar network [14,28,29]. Consistent with this, Ugwoke et al. reported a higher forward-to-backward second harmonic generation ratio in the TLF than in the fascia lata (TLF = 0.53; FL = 0.40), reflecting intrinsic differences in higher-order collagen architecture [33]. It is already reported that TLF works as a large proprioceptive element, capable of sensing deformations and tensions in multiple directions, as reflected by its innervation distribution: a thin and delicate net of free nerve endings, strongly connected with the ECM and particularly responsive to stretch, shear loading, and mechanical stimuli [34]. Collagen type VI plays a key role in peripheral nerve biology, regulating Schwann cell differentiation, preserving myelination, and maintaining nerve structure and function, as well as orchestrating nerve regeneration after injury [35,36]. In a collagen type VI knockout mouse model, the absence of collagen type VI leads to structural myelin alterations, reduced nerve conduction velocity with shorter internodal length, and impaired motor coordination. Sensory function is also affected: *Col6a1*^−/−^ mice exhibit disorganized C-fibers and delayed nociceptive responses to thermal and mechanical stimuli [37]. Our results, showing high collagen type VI levels in both superficial and deep lumbar fasciae, support the idea that this region serves as a key proprioceptive and sensory element, responsive to tactile, mechanical, and stretch stimuli.

The fascia lata, on the other hand, is subjected to greater but more consistently directed mechanical stress [38,39]. As a result, we can assume that it requires a greater proportion of collagen types that provide mechanical strength, such as collagen type I [17].

## 4. Materials and Methods

### 4.1. Tissue Collection

Superficial and deep fascia samples (approximately 1 cm^2^) were collected from adult patients (ages from 20 to 83 y/o; 6 females, 13 males). A total of 19 superficial fascia samples (mean age 54 ± 19 years) and 12 deep fascia samples (mean age 54 ± 20 years) were obtained, since deep fascia could not be collected in all cases (Table 1). Eight samples were collected from the lumbar region and eleven from the thigh during elective surgeries at the Orthopedic Unit of the University of Padova Medical Center (Table 1). The collection of human fascial tissue was approved by the Ethics Committee of the University Hospital of Padova (approval no. 3722/AO/16, approved on 21 April 2016). The research was performed in accordance with the ethical standards of the 1964 Declaration of Helsinki as revised in 2000 and those of Good Clinical Practice. All subjects participating in the study gave their oral and written informed consent.

After collection, all specimens were immediately transferred to the Institute of Human Anatomy, Department of Neuroscience, University of Padua, for histological and biochemical analyses.

The inclusion criteria for participation consisted of these parameters: adult patient (age > 18 years); male or female; patients who underwent emergency surgery for spinal trauma; and patients who underwent femoral head replacement. The exclusion criteria included known connective tissue disorders; any indication of altered collagen composition or systemic conditions affecting collagen metabolism; active malignancy; chronic lumbar spinal pathology; diagnosed diabetes; and tumors.

Each sample was divided into two pieces. One portion was frozen at −80 °C for immunoblotting and hydroxyproline content analysis, while the second piece was fixed in 10% buffered formalin solution for histological and immunohistochemical analyses. Fixed samples were dehydrated in graded ethanol and xylene, embedded in paraffin, and cut into 5 µm thick sections by Leica RM2255 microtome. Dewaxed sections underwent hematoxylin–eosin, Azan Mallory, and immunohistochemistry.

### 4.2. Hematoxylin and Eosin Staining

Hematoxylin and eosin (H&E) staining was performed to evaluate the general morphology of the tissue. Deparaffinized sections were stained in hematoxylin (4 min) to stain nucleic acids and then counterstained with eosin (1 min) for the cytoplasm. After being dehydrated through an ascending alcohol series and cleared in xylene, the samples were finally mounted with Eukitt (Agar Scientific Elektron Technology, Stansted, UK).

### 4.3. Azan Mallory Staining

Azan Mallory staining was performed to evaluate the fascia ECM, with particular focus on connective tissue organization and collagen fiber distribution. After deparaffinization and rehydration, tissue sections were incubated in azocarminium solution containing 1% acetic acid (freshly added before use) for 60 min at 60 °C, followed by rinsing in deionized water to stop the reaction. Slides were then immersed in acid alcohol for 1 min and rinsed for 5 min in deionized water. Sections were subsequently stained with phosphotungstic acid for 5 min, briefly rinsed again in deionized water, and finally immersed in Mallory solution until optimal color intensity was reached. The reaction was terminated by rapid washing in deionized water. Slides were then dehydrated, cleared, and mounted with Eukitt (Agar Scientific Elektron Technology, Stansted, UK).

### 4.4. Immunohistochemistry

Dewaxed sections were stained for collagen type I, collagen type III, collagen type VI, and collagen type XII. For collagen type I staining, the slices were incubated in EDTA solution (Ethylenediaminetetraacetate—Sigma Aldrich, St. Louis, MO, USA) pH 9, at 90 °C for 15 min, to permit the antigen retrieval, and then washed in PBS. All the sections were treated with 3% H_2_O_2_ in PBS for 15 min to inhibit endogenous peroxidases. After washing in PBS and a 1 h incubation in blocking solution (PBS + 0.2% bovine serum albumin (BSA)), samples were incubated overnight at 4 °C with the following primary antibodies: Anti-collagen I in Goat (Southern-Biotech, 1:400, Birmingham, AL, USA); Anti-collagen III in Mouse (Abcam, 1:350, Cambridge, UK); Anti-collagen VI in Rabbit (Proteintech, 1:500, Rosemont, IL, USA); Anti-collagen XII in Mouse (Santa Cruz Biotechnology, 1:40, Dallas, TX, USA).

After repetitive PBS washing, the sections were then incubated for 1 h in PBS + 0.2% BSA with the secondary antibodies HRP-conjugates (Horseradish Peroxidase): Rabbit Anti-Goat (Jackson ImmunoResearch (West Grove, PA, USA), 1:300); Goat Anti-Mouse (Jackson ImmunoResearch, 1:500); Goat Anti-Rabbit (Jackson ImmunoResearch, 1:250).

The reaction was then developed with 3,3′-diaminobenzidine (Liquid DAB + substrate Chromogen System kit Dako), stopped with distilled water, and counterstained with Toluidine Blue 0.1%. All images were acquired by Leica DMR optical microscope (Leica Microsystem, Wetzlar, Germany).

### 4.5. Hydroxyproline Assay Kit

The quantity of hydroxyproline (HYP) in fascial samples was evaluated using a colorimetric assay (MAK008, Sigma-Aldrich), as an indirect indicator of collagen content, being a major component of collagen and largely restricted to it.

In brief, about 50 mg of tissue samples was homogenized using 100 μL H_2_O for every 10 mg of tissue. Then, 100 μL of tissue homogenate was transferred to a pressure-tight vial; 100 μL concentrated hydrochloric acid (10 N) was added and hydrolyzed at 120 °C for 3 h. In total, 10 μL of supernatant was transferred to a 96-well plate and allowed to evaporate in oven at 60 °C. Then 100 μL of the chloramine T/oxidation buffer mixture was added to each sample and incubated at room temperature for 5 min. HYP concentration was determined by the reaction of 100 µL of oxidized HYP with 4-(dimethylamino) benzaldehyde (DMAB), resulting in a colorimetric product. The absorbance was measured at 560 nm using the VICTOR-3™ automated microplate reader (Perkin Elmer, Waltham, MA, USA) and converted into µg of hydroxyproline/mg of tissue, based on the standard curve obtained with the collagen standard solution (from 0 to 1 µg/µL).

### 4.6. Western Blot

Immunoblotting quantified the collagen fibers of different types: I, III, VI, and XII.

Briefly, the frozen samples were thawed, cut with a surgical scalpel, and mechanically digested; total proteins were extracted using RIPA lysis buffer (Thermo Scientific, Waltham, MA, USA) and quantified with the BCA Protein Assay Kit (Thermo Scientific). Equal amounts of proteins (20 µg) were separated on precast polyacrylamide gel with a gradient of 4–25% (Mini-PROTEAN^®^ TGX™ Precast Gels, Bio-Rad, Hercules, CA, USA) and transferred onto polyvinylidene difluoride membranes, PVDF (Bio-Rad). The membranes were incubated with a blocking solution for 1 h at room temperature, and then incubated overnight at 4 °C with the corresponding primary antibodies as follows:

- Anti-collagen I in Goat (Southern Biotech, 1:1000) in TBS (Tris-buffered saline solution) + 5% non-fat dry milk, blocking solution TBS + 5% non-fat dry milk;
- Anti-collagen III in Rabbit (Abcam, 1:6000) in 0.5% BSA in PBS, blocking solution PBS + BSA 4%;
- Anti-collagen VI in Rabbit (Proteintech, 1:2000) in PBS + 2% non-fat dry milk, blocking solution PBS + 5% non-fat dry milk;
- Anti-collagen XII in Mouse (Santa Cruz, 1:1000) in 2% BSA in PBS, blocking solution PBS + 5% non-fat dry milk.

After repetitive washes in PBS or TBS, the membranes were incubated, respectively, with goat anti-rabbit–RP (Jackson ImmunoResearch, 1:5000), rabbit anti-goat (Jackson ImmunoResearch, 1:12,000), or goat anti-mouse (Jackson ImmunoResearch, 1:5000) antibody for 1 h at room temperature.

After repetitive washes, the immunoreactive reaction was determined by SuperSignal™ West Pico PLUS Chemiluminescent Substrate (Thermo Scientific). The intensity of the bands was measured in ATOM UVITEC (Uvitec, Milan, Italy). Images were analyzed with the Q9 Alliance Software (Uvitec, Milan, Italy) and normalized on the same membrane based on the total protein amount transferred to the membrane, evaluated with Ponceau-S staining [40] and subsequent analysis with ImageJ Software 1.54p (Analyze Gel–Plot Lanes) (freely available at http://rsb.info.nih.gov/ij/, accessed on 10 November 2025). Each protein (collagen I, collagen III, collagen VI, and collagen XII) was analyzed in all the samples at least in duplicate. The original blots with labels are provided in the Supplementary Materials. 

### 4.7. Statistical Analysis

All the results were obtained from at least three independent replicates for each experiment and are presented as the mean ± standard deviation. The GraphPad Prism 3.0 statistical package (GraphPad Software Inc., San Diego, CA, USA) was used for the analysis. The results were tested by one-way analysis of variance followed by Tukey’s test for multiple comparisons, and non-parametric unpaired *t*-test (Mann–Whitney U test).

* *p* < 0.05; ** *p* < 0.01 were considered the limits for statistical significance.

## 5. Conclusions

**Figure 8:**
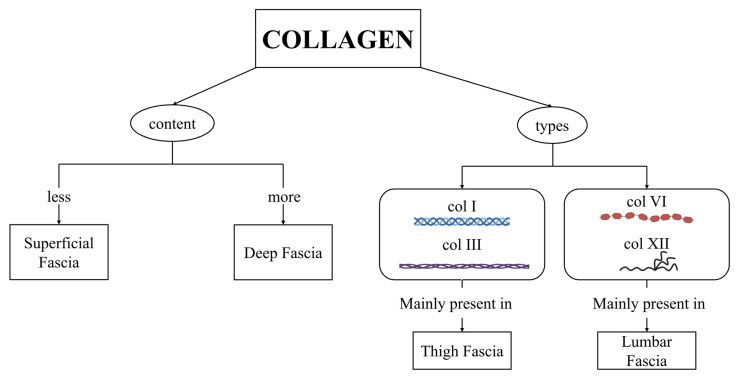
Summary diagram: deep fascia has a higher amount of total collagen with respect to the superficial one. Both fasciae have collagen types I, III, VI, and XII, which display different structures [17,19,24]. Fasciae of the thigh and of the lumbar area display distinct collagen profiles: collagen type I and III are mainly present in the fascia of the thigh, while collagen type VI and XII are mainly present in the fascia of lumbar region.

In conclusion, this study reveals that human fasciae contain not only collagen types I and III but also substantial amounts of collagen types VI and XII, with collagen VI emerging as the predominant subtype with a diffuse distribution. Fasciae of the thigh and the lumbar region displayed distinct collagen profiles, likely reflecting different mechanical roles (Figure 8).

These findings highlight the structural complexity and regional specialization of fascia. Notable variability is observed not only between superficial and deep layers but also across different anatomical sites. Importantly, it was reported for the first time the significant presence of collagen types VI and XII in human fasciae, representing a novel and highly relevant finding. The cohort of patients was heterogeneous in age and sex, according to specific inclusion/exclusion criteria that avoided the recruitment of individuals with known collagen pathologies. Moreover, recruitment from a public hospital limits the selection of an “ideal” population, which we acknowledge as a study limitation. However, the small sample size limits further sub-categorization by factors such as sex, age, or other variables, which showed no significant differences between subgroups. Nevertheless, it is evident that there is considerable interpersonal variability in the amount of the various collagens and in fascia composition, which may result in differences in rigidity, mobility, and pain perception among individuals. Studies with larger cohorts will be essential to elucidate the variabilities and to identify the stimuli driving modifications in the collagen matrix. Moreover, future investigations should potentially consider other collagen types, which have yet to be identified in fasciae. As research progresses, the complexity of fascia organization becomes increasingly evident. 

## Figures and Tables

**Table 1:** Age (y/o: years old), sex (M: male; F: female), and area of collection (Thigh, Lumbar) of the samples of superficial and deep fasciae, with the mean age ± standard deviation for both the groups.

		Superficial Fascia	Deep Fascia
Sample	Area	Age, Sex	Age, Sex
1.	Thigh	31 y/o, M	-
2.	Thigh	32 y/o, M	-
3.	Thigh	54 y/o, M	-
4.	Thigh	63 y/o, F	-
5.	Thigh	64 y/o, F	64 y/o, F
6.	Thigh	65 y/o, F	-
7.	Thigh	68 y/o, M	-
8.	Thigh	70 y/o, F	-
9.	Thigh	77 y/o, F	77 y/o, F
10.	Thigh	81 y/o, M	81 y/o, M
11.	Thigh	83 y/o, M	83 y/o, M
12.	Lumbar	20 y/o, M	20 y/o, M
13.	Lumbar	22 y/o, F	22 y/o, F
14.	Lumbar	33 y/o, M	33 y/o, M
15.	Lumbar	49 y/o, M	49 y/o, M
16.	Lumbar	52 y/o, M	52 y/o, M
17.	Lumbar	52 y/o, M	52 y/o, M
18.	Lumbar	53 y/o, M	53 y/o, M
19.	Lumbar	73 y/o, M	73 y/o, M
		***Mean age 54* *±* *19***	***Mean age 54* *±* *20***

## Data Availability

The original contributions presented in this study are included in the article and Supplementary Material. Further inquiries can be directed to the corresponding author.

## Supplementary Materials

The following supporting information can be downloaded at: https://www.mdpi.com/article/10.3390/ijms27021021/s1.
