# Supplementary material for: Redefining the Collagen Composition of Human Fasciae: Emerging Collagen Types and Structural Heterogeneity
Source: Int J Mol Sci. 2026 Jan 20;27(2):1021. doi: 10.3390/ijms27021021 (PMC12842560; doi:10.3390/ijms27021021)
Supplement: Supplementary file 1 [file ijms-27-01021-s001.zip › ijms-4082342-supplementary.pdf]

PONCEAU-S

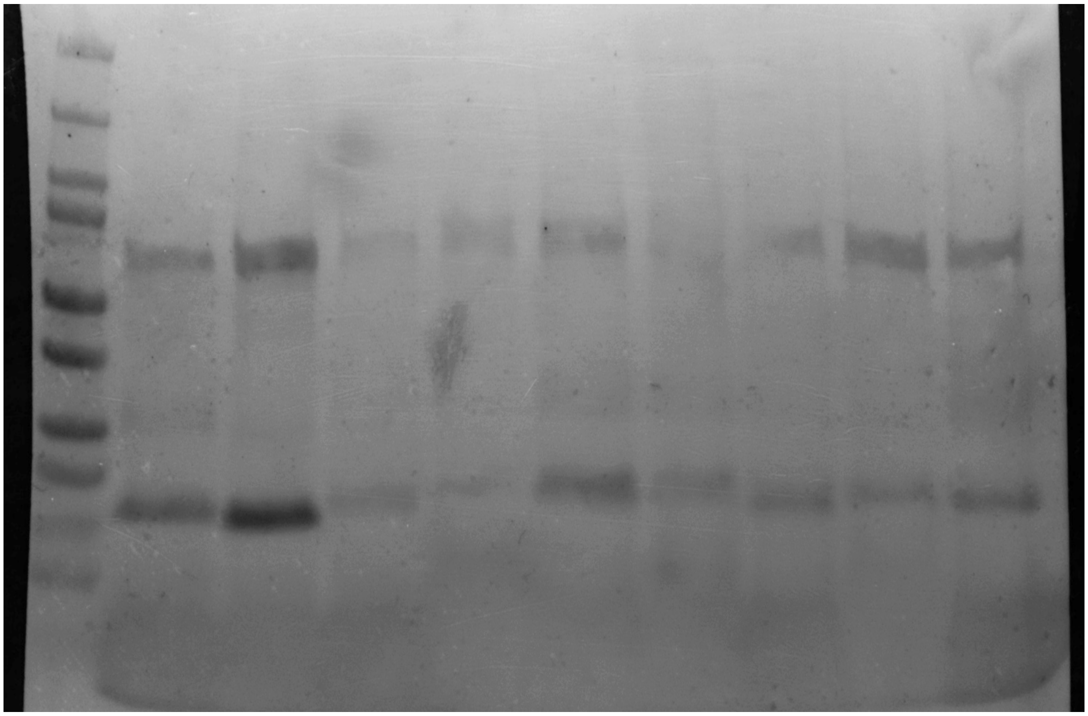

COLLAGEN TYPE I

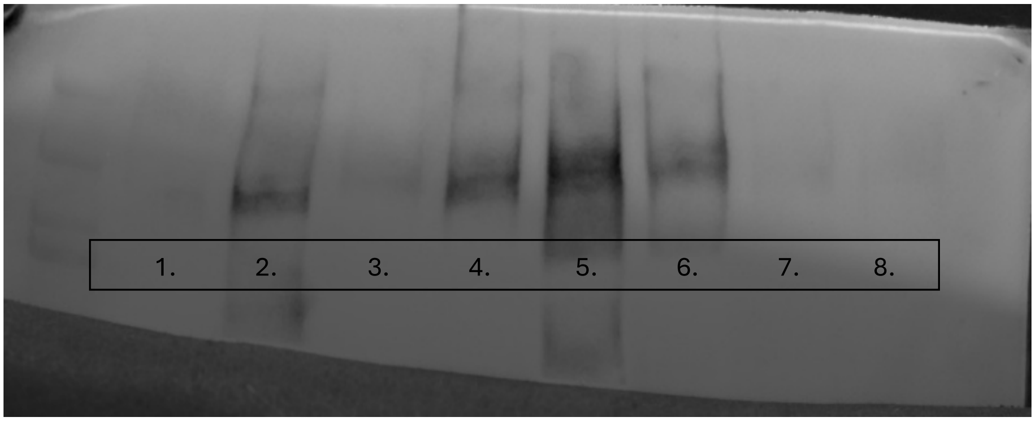

1. Lumbar Superficial F.
2. Lumbar Deep F.
3. Lumbar Superficial F.
4. Lumbar Deep F.
5. Lumbar Superficial F.
6. Lumbar Deep F.
7. Lumbar Superficial F.
8. Lumbar Deep F.

COLLAGEN TYPE III

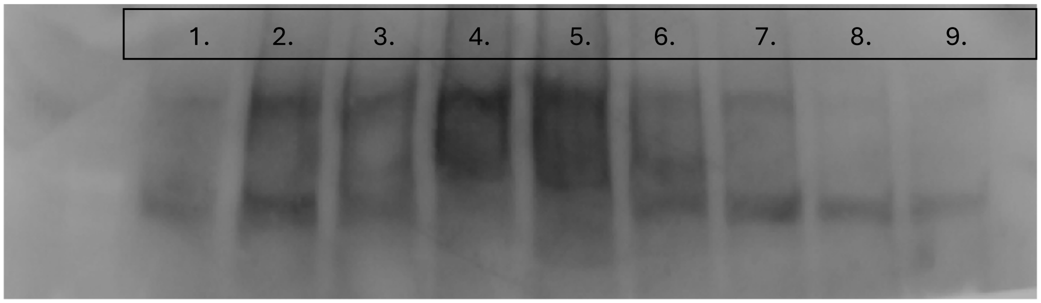

1. Lumbar Superficial F.
2. Lumbar Deep F.
3. Lumbar Superficial F.
4. Lumbar Deep F.
5. Lumbar Deep F.
6. Lumbar Superficial F.
7. Lumbar Deep F.
8. Lumbar Superficial F.
9. Lumbar Deep F.

COLLAGEN TYPE VI

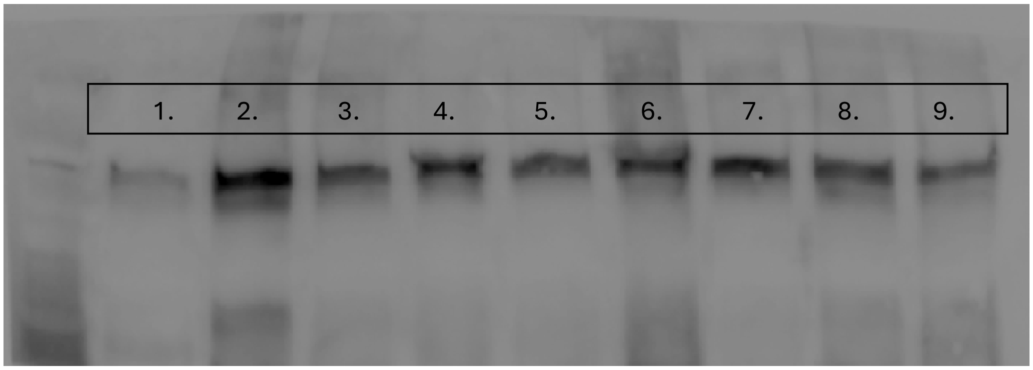

1. Lumbar Deep F.
2. Lumbar Deep F.
3. Lumbar Deep F.
4. Lumbar Deep F. -
5. Lumbar Superficial F.
6. Lumbar Deep F.
7. Lumbar Superficial F.
8. Lumbar Deep F.
9. Thigh Deep F.

COLLAGEN TYPE XII

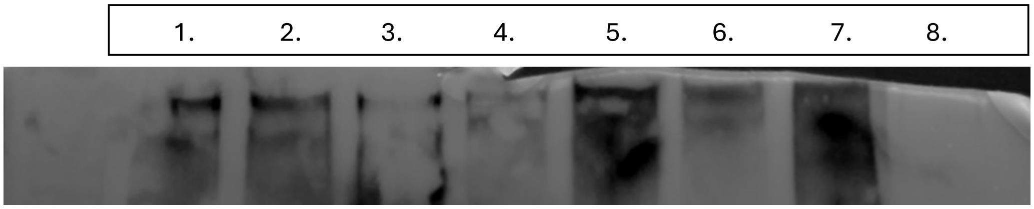

All Lumbar deep fascia
